# Starting Afresh: An Unfinished History

**Published:** 1968-04

**Authors:** G. P. Tonkin


### STARTING AFRESH: AN UNFINISHED HISTORY

G. P. TONKIN, B.SC., V.R.D.

### INTRODUCTION

This paper is an attempt to present a novel set of views to the medical profession; it is a statement of his case by a partially-recovered, brain-injured patient. However, it must be admitted that it is also a mental exercise, forming part of the attempts at self-cure that are doubtless considered essential in such cases.

The injury was a fractured skull due to impact with the windscreen of a car. Unconsciousness for about two months followed, allowing and helping the slow piecing together of the cranium and most of its contents. Then there were about two more months in hospital, though it was more than 18 months before return to work was permitted. The slow improvement so far seems to follow a linear gradient, and if this is maintained it seems reasonable to forecast a five-year recovery period; the main need is patience.

### DETAIL

The patient is a factory programme engineer, on duty as a Lieutenant Commander R.N.R., who at the age of 41 was serving his annual training in H.M.S. Venturer, which called at Oporto, Portugal. He had apparently intended to stay aboard on 17th August, 1965, but two officers of H.M.S. Thames alongside had hired a self-drive car and offered him the front passenger seat, which he accepted. The car had the misfortune to have a fairly high-speed impact with a lamp post. There were no seat-belts, with the result that the guest was thrown against the windscreen, through which his head smashed a hole. The driver and the other passenger were rendered unconscious at the same time, but without fractured skulls because their impacts were broken, in the driver's case by the steering wheel and in the rear passenger's case by his remoteness.

The patient was taken to the public hospital in Oporto, which has the widest range of facilities. There he was in the hands of a capable Portuguese surgeon who displayed firm hopes for his long-term recovery. The patient's wife had been flown straight out, and remained in Oporto until he was well enough to be flown home.

All of this period is now a blank to the patient, as well as the time he spent in various British hospitals, until he at last returned to his home in Bristol just before Christmas 1965; and then followed a very slow work-up which has not yet been completed. The specialist had advised that continuous mental probing and searching was the most effective cure, but it may be said that failure to understand the situation has been one of the greatest difficulties of the patient, causing much tribulation and hopelessness. But frequently attempts of this kind forced the brain into action, and resulted in some " connections " being re-formed.

One of the interesting features noticed by the patient has been the infuriation engendered by his attempts to return to work. At first there was blank failure to understand aspects of the job he had personally created, and this resulted in a feeling of failure in 1966. The re-beginning was ultimately made possible by starting in a very junior position at the end of January 1967, and slowly working up. This was a considerable tax on the patient's pride, which has had to be subjugated. Now, however, full time has been achieved, though many normally automatic actions must still be laboriously worked out through the networks we normally take for granted.

The patient finds it a somewhat curious circumstance that as his comprehension increases the situation appears not even to be the same, but actually worse, and there is much tiring worrying over trifles. In fact return of brain power has seemed a depressing circumstance, because standards appear to rise more quickly than ability; but this sort of apparent affliction must be accepted as part of the recovery.

Failure of memory may be due to a loss of brain cells, though it appears more likely to be due to loss of automatic access equipment. Thus a careful slow reminder will often bring back an unsuspected item that appeared to have gone for ever. There has also been some evidence of a reduction in the number and capacity of brain routes, resulting in temporary inability to bring a number of facts or circumstances together for reasoning. Time is wasted in trying to remember what would be the best course of action, or what one had decided to do in a particular situation.

Vocabulary and grammar have recovered early but are recognised as a " failure ", and there is a frequent searching for words, which obstructs communication*. Handwriting is another interesting detail. Let us admit that it was always somewhat irregular; but additional faults now occur. The differences in speed between the various brain channels make hand movement overtake thought, causing mis-spellings, and there are frequent incorrect words. Recognition of this fault makes one reluctant to write, or to carry out any operation in which one distrusts one's ability; but in fact the only way to combat such brain sluggishness is to accept the situation and " press on ".

Another grave difficulty is the tendency of the person to feel that he is written off as now incapable. Few people realise the time scale of recovery from the injury, and how the same slow rate of continuous progress has gone on for $2\frac{1}{2}$ years since the accident. There has also been a failure by the patient to realise that his family are bound to be seriously affected by the long period of their ministrations. A tendency exists for him to want to be isolated, fearing the rate of normal happenings. But no progress can be expected in that way; he must expect several years of hard, up-hill mental work.

There was an initial false assumption by the patient that he could just drop back into place with many things, such as driving a car. The specialist rightly insisted that he should take the driving test again. The reasons were not appreciated at the time, but the lessons were laboriously accepted, though with some reluctance, after 23 years of fault-free driving. The test was passed,

*Some, but little, re-phrasing has been needed in preparing this essay for publication, on this account.—Editors.

though the fact that two attempts were needed was taken as an insult! Relief followed, with the assumption that all had returned. Then there occurred a misjudgement of conditions that caused an accident—not serious, but sufficient. There was then the realisation that going to the bottom of the ladder had been necessary, and that one had to exercise great care under all conditions, just as a learner-driver has. How tortuous the methods that must be adopted to get through many "common" actions!

### ANALYSIS AND CONCLUSION

The brain damage was not apparently to the actual computing mechanism, but to the storage cell system or memory, and to the input/output mechanisms. Explanation of this to the patient after a mental hospital examination was most instructive, bringing new hope. It cannot be emphasised enough that encouragement is one of the main necessities for cure in such cases. It is also clear that symptoms must vary enormously for different places of brain injury, and it is unlikely that the injured will have sufficient comprehension to contribute much himself.

It seems that we must see this injury as brought about partly by actual loss of cells and partly by disconnection. The disconnected areas can be reconnected by personal effort in many cases, and though they may not apply themselves immediately to some circumstance, even of familiar nature, the slow process of acceptance of a new "idea" is at last effective. The disconnection of cells that act as a data store is a most serious matter, in that memory is severely reduced. But so also is the damage to the input/output mechanism, which gives rise to an inability to carry out complicated manœuvres that are normally a matter of course.

An interesting point is that the very progress of brain recovery brings increasing awareness of one's disabilities, and of the fact that improvement in some faculties is delayed, so that one gets the impression of an actual worsening of health. The patient naturally expects that his recovery should proceed equally in all his mental processes, but this seems not to be so. He could more cheerfully accept these disappointments if he were warned that the time-scale of recovery will be longer for some faculties than for others.

The main need in this type of case is to restore the confidence of the patient, giving him copious time and lack of worry, and a sense of achievement. I am sure the evidence of a layman will be tolerated; it is not contended that this is by any means a complete analysis, but it is put forward as a small contribution to the help of the similarly injured. Clearly, says the partly recovered, there is no reason why recovery should not be complete, if a larger time-scale than he first thought likely is accepted.

It is essential that the brain be used and practised to assemble facts, thus restoring the confidence and ability of the patient. I beg to assure readers that I believe that Almighty God plays the major part in these things. If some sort of appeal from the patient is permitted, please be optimistic even though one's statements could be made safer by being pessimistic. Hope is one of the governing factors to the injured.

